# Prognostic role of simplified Pulmonary Embolism Severity Index and the European Society of Cardiology Prognostic Model in short- and long-term risk stratification in pulmonary embolism

**DOI:** 10.12669/pjms.306.5737

**Published:** 2014

**Authors:** Talat Kilic, Hakan Gunen, Gazi Gulbas, Suleyman Savas Hacievliyagil, Ali Ozer

**Affiliations:** 1 Talat Kilic, Department of Pulmonary Medicine, Inonu University Faculty of Medicine, Turgut Ozal Medical Center, Malatya, Turkey.; 2 Hakan Gunen, Department of Pulmonary Medicine, Training and Research Hospital of Ministry of Health, Istanbul, Turkey.; 3 Gazi Gulbas, Department of Pulmonary Medicine, Inonu University Faculty of Medicine, Turgut Ozal Medical Center, Malatya, Turkey.; 4 Suleyman Savas Hacievliyagil, Department of Pulmonary Medicine, Inonu University Faculty of Medicine, Turgut Ozal Medical Center, Malatya, Turkey.; 5 Ali Ozer, Department of Public Health, Inonu University Faculty of Medicine, Turgut Ozal Medical Center, Malatya, Turkey.

**Keywords:** Mortality, Pulmonary embolism, Prognostic model, Risk assessment

## Abstract

***Objectives: ***Hemodynamic status, cardiac enzymes, and imaging-based risk stratification are frequently used to evaluate a pulmonary embolism (PE). This study investigated the prognostic role of a simplified Pulmonary Embolism Severity Index (sPESI) score and the European Society of Cardiology (ESC) model.

***Methods*** ***:*** The study included 50 patients from the emergency and pulmonology department of one medical center between October 2005 and June 2006. The ability of the sPESI and ESC model to predict short-term (in-hospital) and long-term (6-month and 6-year) overall mortality was assessed, in addition to the accurancy of the sPESI and ESC model in predicting short-term adverse events, such as cardiopulmonary resuscitation, or major bleeding.

***Results*** *:* Of the 50 patients, the in-hospital and 6-year mortality rates were 14% and 46%, respectively. Fifteen (30%) of these experienced adverse events during hospitalization. Importantly, patients classified as low-risk according to the sPESI had no short-term adverse events as opposed to 4.8 % in the ESC low-risk group. They also had no in-hospital, 6-month, or 6-year mortality compared to 4.8%, %14.3, and %23.8, respectively, in the ESC low-risk group.

***Conclusions:*** The sPESI predicted short-term and long-term survival. The exclusion of short-term adverse events does not appear to require imaging and laboratory testing.

## INTRODUCTION

Acute pulmonary embolism (PE) is related to high (4 to 13%) short-term (in-hospital or 30-day) mortality rates.^1^^,^^2^ Recent, studies have revealed that PE heralds an increased long-term risk of adverse outcomes after hospital discharge with 1-year mortality rates as high as 25%.^3^^-^^6^ Early PE-related mortality is associated with clinical results and underlying disease.^2^ As several prognostic models have limitations in daily clinical practice, a few have been recommended for risk stratification in acute PE.^7^^-^^11^ The Pulmonary Embolism Severity Index (PESI) is one of the most widely validated prognostic models for 30-day mortality.^9^ Studies have demonstrated that this model can identify patients with a low mortality risk who may be treated as outpatient.^12^^,^^13^ However, the PESI may not be suitable for routine clinical practice in busy emergency or pulmonology departments, as it requires the calculation of a score based on many different variables, and each parameter has a diverse value. Recently, Jimenez et al.^14^ proposed a simplified PESI (sPESI). As shown in Table-I, the sPESI contains only seven variables. In a previous study, the sPESI had similar accuracy in predicting short-term mortality in PE patients and offered great ease of use.^14^

In the last decade, the presence of shock, persistent arterial hypotension, and signs of acute right ventricular dysfunction (RVD) based on imaging or biomarkers have been used as prognostic indicators of PE.^15^ The prognostic model of the European Society of Cardiology (ESC) categorizes patients into high-, intermediate- or low-risk groups based on these findings.^6^

The majority of studies have used the sPESI and ESC prognostic model to predict 30-day or 90-day mortality after acute PE.^9^^,^^15^ To our knowledge, only one study evaluated the relationship between the sPESI and 6-month mortality.^16^ Likewise, a limited number of studies have compared the sPESI and ESC model.^7^

The present study assessed the accuracy of the sPESI and ESC prognostic model in predicting short-term (in-hospital) adverse events and short-and long-term mortality in PE patients. In addition, two prognostic models were compared in terms of predict short- and long- term prognosis.

## METHODS

***Study design*** *:* Prospective baseline data collected from the time of PE diagnosis and outcome data from the same cohort were used to determine the ability of the sPESI and ESC prognostic model to predict in-hospital adverse events (including in-hospital mortality), 6-month and 6-year overall mortality. The sPESI score for each patient was retrospectively calculated based on the criteria shown in Table-I. Patients were classified as low-risk (0 points) or high-risk (≥1 point).^14^ In the ESC prognostic model, high-risk patients were identified by the presence of shock or persistent arterial hypotension; intermediate-risk patients were classified according to the presence of RVD based on echocardiography and/or elevated Cardiac TroponinI (cTnI) levels; and low-risk patients were categorized as those having none of the aforementioned sign and symptoms.^6^ Intermediate- and high-risk patients were combined in a single category called the elevated risk group.^7^ The proportion of low-risk vs. elevated-risk patients based on the sPESI and ESC model was calculated, in addition to the proportion of patients with in-hospital adverse events (including overall mortality), 6-month and 6-year overall mortality in the low-risk vs. elevated-risk groups. Informed consent was obtained from all the patients. The local ethics committee approved this study (No: 2005/94) 

***Patients and setting:*** Patients were included from the emergency and pulmonology department of Turgut Ozal Medical Center between October 2005 and June 2006. The diagnosis of PE was confirmed either by contrast enhanced computerized homographic pulmonary angiography according to previously described criteria,^17^ a high-probability ventilation–perfusion scan result^18^ or lower limb venous compression ultrasonography positive for proximal deep vein thrombosis in patients with inconclusive ventilation–perfusion scans.^19^

***Echocardiography Examination and cTnI: ***Transthoracic echocardiography and cTnI testing was performed within the first 24 hour of PE diagnosis in all patients. RVD was confirmed as paradoxical septal motion, hypokinesis of the RV-free wall, and right ventricular dilatation (end-diastolic diameter >30 mm or right-to-left ventricular end-diastolic diameter >1 mm on the apical 4-chamber view), and pulmonary hypertension.^20^ In this study, cTnI concentrations of ≥0.1 ng/mL **were** an indication of myocardial injury.

***Study outcomes:*** The primary outcome used to validate the prediction rules was in-hospital mortality and adverse events after diagnosis of acute symptomatic PE. In-hospital adverse events were ≥1 of the following: need for thrombolytic treatment, catecholamine support of blood pressure (except for dopamine infused at a rate of ≤ 5 μ g kg^-1^ min^–1^), endotracheal intubation, cardiopulmonary resuscitation, major bleeding, and symptomatic recurrent venous thromboembolism (VTE). Overall mortality was defined as death from any causes. Bleeding complications were classified as major if they were overt and were either associated with a decrease in hemoglobin level of ≥2.0 g dL^-1^, required a transfusion of ≥2 unit of blood, or were retroperitoneal or intracranial. Hospital records were used to determine in-hospital mortality and adverse events. Data on overall 6-month and 6-year mortality were obtained from the national death registration system. 

***Treatment: ***All the patients were treated according to current guidelines.^6^

***Statistical analysis:*** General characteristics of the patients are presented as mean ±SD for continuous data and* n* (%) for categorical data. Most continuous variables were dichotomized, and the proportions in each group are described. The analysis used the x^2^ or Fisher’s exact tests to compare categorical data between groups. Continuous variable were compared with the Kruskal-Wallis test. The McNemar test was used to compare the proportions of patients with adverse events between groups. The accuracy, sensitivity, specificity, positive predictive value (PPV), negative predictive value (NPV), positive likelihood ratio (PLR), and negative likelihood ratio (NLR) of both models were calculated. 

## RESULTS

Fifty (48%, 24 men) consecutive patients with confirmed diagnosis of acute PE who agreed to participate in the study were enrolled. CT pulmonary angiography was most frequently used to confirm acute PE (n = 34, 68%). Table-II shows patients’ clinical symptoms, predisposing conditions, and relevant findings at presentation.

Fifteen (30%) patients experienced at least one in-hospital adverse events. Of these, seven (46.7% of all adverse events) patients died during hospitalization. Of these, five (71% of all hospital deaths) died of PE-related, one died as a result of major bleeding, and another died because of multiple metastatic lung cancer. Overall, in the 6-year period, 23 patients died. Of these, seven patients died in hospital, and 12 patients died within 6-months after discharge. The other four deaths occurred within or after 6 months. In the current study, the sPESI classified lower proportion of patients as low risk (38% [19 of 50]) compared to ESC prognostic model (42% [21 of 50],* P*.05). Importantly, there were no in-hospital adverse events in the sPESI low-risk patients as opposed to 4.8% (1 of 21) adverse events in the ESC low-risk group (Table-III). In the latter case, the patient died in the hospital as a result of multiple metastatic lung cancer. In addition, there were no in-hospital, 6-month, or 6-year mortality in the sPESI low-risk patient as opposed to 4.8% (1 of 21), 14.3% (3 of 21) and 23.8% (5 of 21), respectively, in the ESC low-risk group. At the other ends of the severity spectrum, the sPESI high-risk patients had slightly higher in-hospital mortality (22.6%) compared with the ESC elevated-risk patients (20.7%; *p>*0.05). On the other hand, high-risk patients based on the sPESI had higher 6-month and 6-year overall mortality compared with the ESC elevated-risk patients (Table-III).

As shown in Table-IV, the sPESI had higher sensitivity, a higher NPV, and a lower NLR than the ESC model for predicting in-hospital mortality in the study cohort. When all in-hospital adverse events were considered, the NPV of the sPESI for predicting low risk was 100% compared with 95.2% for the ESC prognostic model. Thus, the sPESI appeared to be more accurate than the ESC model in excluding short-term adverse events, including in-hospital overall mortality, and long-term overall mortality. Interestingly, the specificity, PPV, and PLR of the sPESI were higher than those of the ESC model in predicting 6-month and 6-year overall mortality (Table-IV).

## DISCUSSION

In the current study, both the sPESI and ESC prognostic model successfully predicted short-and long-term mortality, but short-term adverse events could safely be excluded using the sPESI, without the need for imaging tools or biomarkers such as cTnI. 

After diagnosis, the management of patients with PE is extremely important for risk stratification and decision making regarding therapy. In the last decade, numerous prognostic markers have been evaluated in clinical practice, from several clinical parameters^9^^,^^11^ to RV function assessment,^21^ and plasma determination of B-type natriuretic peptide (BNP) and cTnI.^16^^,^^22^^,^^23^ Clinical prognostic models were developed to recognize low-risk patients with PE who may be candidates for outpatient therapy or a shorter hospital stay.^9^^,^^11^^,^^24^ Some previous studies reported on the prognostic validity of the sPESI in assessing the severity of the disease according to the patient’s co-morbidity and initial clinical findings gathered during the evaluation of PE.^9^^,^^14^ Of these, the hemodynamic status at admission is the most important prognostic factor in patients with acute PE. In particular, in the subgroup of initially normotensive patients with acute PE, the main focus for fast and accurate risk stratification is on RVD or damage to the myocardium caused by acute pressure overload.^8^ Therefore, the ESC guidelines recommended that clinicians use indicators of RVD (echocardiography or BNP) and biomarkers of myocardial damage (cTnI or T) in assessing the severity of PE.^6^

**Table-I:** Simplified Pulmonary embolism Severity Index.^14^

**Variable**	**Point**
Age>80 y	1
History of chronic cardiopulmonary disease	1
History of cancer	1
Pulse >110 beats/min	1
Systolic BP<100 mm Hg	1
SaO_2_<90	1

Sao_2_: Arterial oxyhemoglobin saturation.

Patients with 0 point are low risk group and patients with ≥ 1point are high risk group.

**Table-II:** Baseline characteristics of 50 patients with acute symptomatic PE at presentation

**Clinical characteristics**	**All Patients**	**Death (Any Cause) at 6-year**		***P***
		**No (n: 27)**	**Yes (n:23)**	
Median age	58.1± 19,4	47.8±19,0	70.02±11.4	<0.0001
Age>80 years	7(14)	1(4)	6(26)	0.039
Male	24 (48)	11(41)	13(57)	0.39
Risk factors for VTE				
Cancer	12 (24)	4(19)	8(39)	0.09
Surgery	20 (40)	14(52)	6(26)	0.086
Immobility for >4 days	9(18)	2(7)	7(30)	0.062
Previous VTE	6(12)	4(15)	2(9)	0.67
Comorbid diseases				
Chronic lung disease	4(14)	2(7)	5(21)	0.14
Congestive heart disease	8(16)	3(11)	5(21)	0.4
Clinical presentation at admission				
Heart rate≥110 bpm	30(60)	14(52)	16(70)	0.25
SBP<110 mm Hg	29(14.9)	11(41)	11(48)	0.71
Saturation<90	32(60)	12(44)	20(87)	0.02
Cardiac biomarkers				
cTnI>0.1 ng/dl	28(56)	11(41)	17(74)	0.024
sPESI risk classes				
Low- risk	19(38)	19(70)	0	<0.0001
High-risk	31(62)	8(30)	23(100)	
ESC model risk classes				
Low- risk	21(42)	16(59)	5(22)	0.041
Intermediate	16(32)	8(30)	8(35)	
High-risk	13(26)	3(11)	10(43)	
RVD(+)	24(48)	9(33)	15(65)	0.046
RVD(-)	26(52)	18(67)	8(35)	
Treatment modality				
UFH/LMWH	18 / 18(36/36)	9/11(33/48)	9/7(39/30)	
Thromboliysis	14 (28)	7(26)	7(30)	0.38
Length of hospital stay (day)	15.5 ± 7.4	14.9±7.6	16.0±7.0	0.5

Data are given as N (%) or Mean ± SD.* P* value was given for survival and deaths. UFH: Unfractionated heparin; LMWH: low molecular weight heparin.

**Table-III:** In-hospital adverse events and overall mortality, 6-month and 6-year overall mortality based on the sPESI and ECS prognostic model.

	Total Patient (n:50)	In- hospital adverse events (n: 15)	In-hospital overall mortality (n: 7)	6-month overall mortality (n: 19)	6-year overall mortality (n: 23)
sPESI					
Low risk (n%)	19 (38)	0	0	0	0
High risk (n%)	31 (62)	15( 48.4)	7 ( 22. 6)	19(61. 3)	23(74. 2)
*p *value	*0.016*	*<0.0001*	*0.035*	*<0.0001*	*<0.0001*
ESC					
Low risk (n%)	21(42)	1 (4. 8)	1(4. 8)	3 (14. 3)	5 (23. 8)
Elevated risk (n%)	29(58)	14 (48. 3)	6 (20,7)	16 (55. 2)	18 (62. 1)
*p *value	*0.109*	*0 .001*	*0.045*	0.*004*	*0.007*

**Table-IV:** The value of the sPESI and ECS for predicting in-hospital adverse events and in-hospital overall mortality, and 6- month and 6-year overall mortality in patients with acute PE.

	sPESI	ECS model
In-hospital adverse events		
Sensitivity, %	100	93.6
Specificity, %	48.3	57.4
PPV, %	48.3	73.6
NPV, %	100	95.2
PLR	1.97	3.51
NLR	0	0.11
In-hospital overall mortality		
Sensitivity, %	100	85.7
Specificity, %	44.1	46.5
PPV, %	22.5	20.6
NPV, %	100	95.2
PLR	1.78	1.65
NLR	0	0.30
6-month overall mortality		
Sensitivity, %	100	84.2
Specificity, %	61.2	58.4
PPV, %	61.2	55.1
NPV, %	100	85.7
PLR	2.57	2
NLR	0	0.27
6-year overall mortality		
Sensitivity, %	100	78.2
Specificity, %	70.3	59.2
PPV, %	74.1	62.1
NPV, %	100	76.1.2
PLR	3.36	1.91
NLR	0	0.36

In the current study, the sensitivity, NPV, and NLR of the sPESI were superior to those of the ESC prognostic model for both short- and long-term mortality. However, the sensitivity and NPV of the ESC prognostic model in predicting PE-related mortality (in-hospital) were similar to those of the sPESI. A previous study found that the sPESI had higher sensitivity, a higher NPV, and a lower NLR than the ESC model in predicting 30-day mortality.^7^ These findings are consistent with the data in our study. However, Ozsu et al.^25^ demonstrated similar sensitivity and a similar NPD and PPD for the sPESI and ESC model. In common with the finding of an earlier studies,^6^ our results confirm that the ESC model appears to be more suited to recognizing patients at elevated risk of PE-related mortality in the acute period of PE. The sPESI seems to be more suited to identifying patients who are low risk of mortal and non-mortal clinical outcome. As noted in an earlier study,^7^ our finding confirmed that the NPV of 100% for early adverse events, including in-hospital, in low-risk categories makes the sPESI very useful for discriminating patients for outpatient treatment. Interestingly, in our study, the specificity, PPV, and PLR of the sPESI were higher than those of the ESC model in predicting 6-month and 6-year overall mortality.

In addition to effectively predicting PE-related short-and long-term mortality and adverse events (such as major hemorrhage), optimum prognostic models should identify patients requiring intensive care and aggressive therapy, such as treatment with thrombolytic agents. Ideal prognostic models should rule out (or forecast), in the shortest possible time, short- and long-term overall mortality and adverse clinical outcomes following discharge, as well as identify those suited to outpatient therapy.

 Generally, the ratios of patients categorized by the sPESI as having a low clinical risk were lower than those classified as having a low risk by the ESC model. A significant finding in the present study with regard to decision making in clinical practice is that the sPESI accurately identified low-risk patients who required only short-term hospitalization or who could be managed as outpatients. The superiority of the sPESI to other prognostic models is based on a number of factors., First, it is based on clearly defined and simple clinical data commonly obtained on admission. Second, it takes both the clinical severity of acute PE and the load of concomitant disease into account. Third, it does not require the routine use of costly laboratory tests, such as BNP and cTnI, with potentially lengthy turnaround times, or echocardiography procedures, which require time and experience. Finally, sPESI has advanced prognostic accuracy compared with simple clinical prognostic rules such as shock index.^24^

The present study is valuable for a number of reasons. First, to best of our knowledge, this is the first study in the English literature to assess the accuracy of the sPESI and ESC prognostic model in predicting 6-year overall mortality in PE patients. Second, this study demonstrated that the sensitivity and NPV of the sPESI are higher than those of the ESC model in predicting both in-hospital mortality and long-term (6-month and 6-year) mortality. As shown in the Table-IV, this study also demonstrate that the specificity and PPV of the sPESI are higher than those of the ESC model in predicting both 6-month and 6-year overall mortality. 

The current study has some limitations. First, the main limitation is the small sample size. Second, we could not determine the exact cause of death in the six years after discharge. Therefore, we evaluated patients only in terms of overall mortality. Finally, although we used prospectively collected clinical data, the sPESI and the ESC were performed retrospectively.

## CONCLUSIONS

In conclusion, this study showed that both the sPESI and ESC prognostic model effectively predict short-term and long-term mortality. In particular, sPESI exhibited better prognostic accuracy than the ESC model in predicting low-risk patients with acute PE, without the need for imaging tools or extra laboratory tests. In addition, this study assessed for the first time the accuracy of the sPESI and ESC prognostic model in predicting 6-month and 6-year mortality in acute symptomatic PE patients. According to the data in this study, the patients identified as low risk with the sPESI can be considered for out-of-hospital treatment. 

## Authors Contribution:

**TK:** Contributed to study design, collection of data, analysis and interpretation of data, statistical analysis, critical revision of the manuscript. **HG:** Study design, critical revision of the manuscript. **GG: **Collection of data, analysis, and interpretation of data. **SSH**: Collection of data, analysis, and interpretation of data. **AO: **Statistical analysis.
